# Hemolytic anemia after switching from infliximab originator to biosimilar CT‐P13 in a patient with inflammatory bowel disease: A case report

**DOI:** 10.1002/ccr3.2394

**Published:** 2019-09-21

**Authors:** Anne S. Strik, Geert R. D’Haens, Mark Löwenberg

**Affiliations:** ^1^ Department of Gastroenterology and Hepatology, Amsterdam UMC University of Amsterdam Amsterdam The Netherlands

**Keywords:** gastroenterology and hepatology, hematology, pharmacology, toxicology

## Abstract

Available data on switching from originator infliximab to CT‐P13 in patients with inflammatory bowel disease are reassuring regarding safety and efficacy outcomes. However, monitoring of such patients, especially in the early phase after switching, is important given the possibility that rare side effects might occur, as illustrated here.

## Graphical abstract

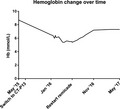

## 1 BACKGROUND

Patent expiration of originator infliximab (IFX) Remicade^®^ has led to the introduction of IFX biosimilar CT‐P13. Although a biosimilar is highly similar to the originator product, subtle changes such as the glycosylation pattern are possible due to the complex molecular manufacturing process. CT‐P13 has demonstrated an equivalent safety and efficacy profile compared with IFX originator in patients with rheumatoid arthritis and ankylosing spondylitis and the European Medicines Agency (EMA) decided to extrapolate these results to other chronic inflammatory diseases, such as inflammatory bowel disease (IBD).^1^, ^2^ Efficacy and safety of CT‐P13 have been demonstrated in IBD patients (ie, ulcerative colitis [UC] and Crohn's disease [CD]) in various clinical trials.^3^, ^4^, ^5^ However, the primary reason to prescribe biosimilars is to reduce healthcare cost, since the price of biosimilars has so far been significantly lower compared with the reference product. Controlled clinical trials are needed in order to investigate the consequences of switching patients from Remicade^®^ to CT‐P13 with regard to both efficacy and safety. The SECURE trial, a multicenter clinical trial performed in the Netherlands and Belgium, investigated pharmacokinetics in patients with quiescent IBD receiving IFX maintenance treatment before and after switching from Remicade^®^ to CT‐P13.^6^ In this noninferiority trial, no differences were observed in IFX serum concentrations at trough after switching to CT‐P13. Noninferiority was also demonstrated for clinical and biochemical disease activity, immunogenicity, and quality of life. In line with these observations, other clinical trials demonstrated similar efficacy, safety, and immunogenicity outcomes.^7^, ^8^, ^9^, ^10^, ^11^, ^12^ Nowadays, switching patients from Remicade^®^ to CT‐P13 has become part of routine care in most European countries since evidence about switching is reassuring.^6^, ^10^, ^12^ Although IFX is an effective therapeutic agent for inducing and maintaining remission in IBD patients, side effects are relatively frequently observed. In the Investigational Medicinal Product Dossier of IFX, hemolytic anemia is reported as a rare side effect (1/10 000 to <1/1000 patients). The underlying mechanism of this phenomenon remains to be characterized. We, here, report for the first time a patient with CD who developed hemolytic anemia 6 months after switching from IFX originator (Remicade^®^) to IFX biosimilar CT‐P13.

## 2 CASE PRESENTATION

A 61‐year‐old male patient was diagnosed with UC in 1996. Due to therapy refractory disease, he underwent a proctocolectomy with ileo‐anal pouch reconstruction in the first year after the diagnosis was made. Postsurgery, the patient was in remission for many years. In 2014, an endoscopy was performed because of pouchitis complaints. The diagnosis was changed to CD based on histopathology findings, and IFX was started in combination with mercaptopurine. After successful induction treatment with Remicade^®^ (5 mg/kg at week 0, 2 and 6), maintenance of remission was achieved and IFX was continued (regular dosing schedule consisting of 5 mg/kg every 8 weeks). The patient was also known with IgA nephropathy, type 2 diabetes, arterial hypertension, and hypercholesterolemia and received treatment with insulin, rosuvastatin, and enalapril. He is a nonsmoker and drinks approximately two alcoholic drinks per day.

In July 2015, he was switched from Remicade^®^ to CT‐P13 in the same dosing regimen. Serial blood samples were taken in order to assess C‐reactive protein (CRP), albumin, IFX trough level, and antidrug antibodies. While continuing treatment with CT‐P13, he maintained in clinical and biochemical remission.

**Figure 1:**
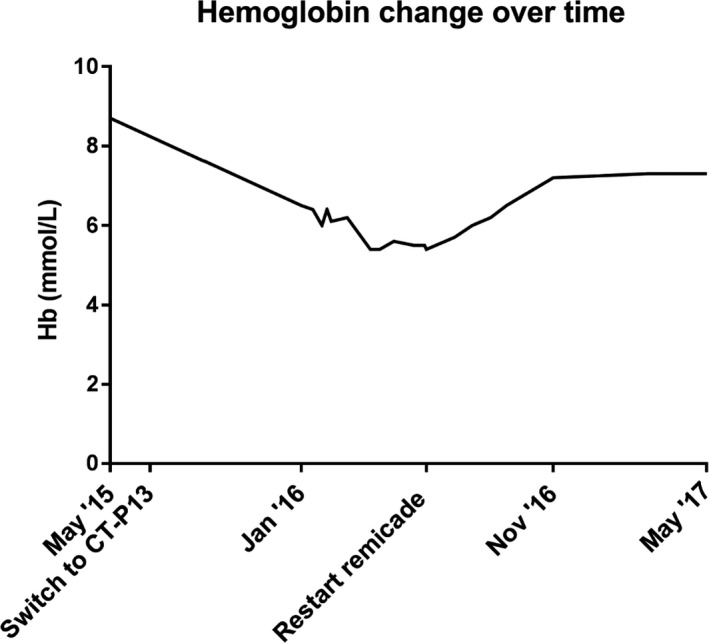
Hemoglobin levels in mmol/L over time. Hb, hemoglobin

In January 2016, 6 months after switching from IFX originator to CT‐P13, he presented at the out‐patient clinic of the Academic Medical Centre with complaints of dyspnoea, fatigue, and chest pain on exertion. Physical examination, as well as electrocardiography, echocardiography, and chest X‐ray, did not reveal any abnormalities. Laboratory assessment revealed a macrocytic anemia with a hemoglobin (Hb) of 10.5 g/dL, compared with a Hb of 14.0 g/dL 7 months earlier (1 month before switching to CT‐P13). The increased mean corpuscular volume (MCV) of 106.7 fL was compatible with 6‐mercaptopurine use. In addition, an increased reticulocyte count of 113.7 × 10^9^/L (reference 25‐75 × 10^9^/L) was found. Serum folic acid and vitamin B12 levels were normal. A Coombs test was performed two times and both negative. Serum haptoglobin was repeatedly unmeasurable (<0.20 g/L; reference 0.3‐2.0 g/L). Antinuclear antibodies (ANA) were positive, and antineutrophil cytoplasmic antibody (ANCA) measurement was negative. A glucose‐6‐phosphate dehydrogenase (G6PD) and pyruvate kinase (PK) deficiency was excluded. The results of the laboratory tests are shown in Table 1. In the next 4 months, the Hb further dropped to 8.7 g/dL. A haematologist was consulted and because of a possible relationship with the switch to CT‐P13, it was decided together with the patient to switch him back to Remicade^®^ in June 2016. The patient was closely monitored at the out‐patient clinic. After switching the patient back to Remicade^®^, an increased Hb was seen over time (Table 1), which was paralleled by clinical improvement. Figure 1 shows the Hb values over time. During this period, the disease remained in clinical remission and his latest endoscopy in November 2017 showed no signs for disease activity. Mercaptopurine was stopped after the endoscopy and he is currently in clinical remission while receiving IFX monotherapy.

**Table 1:** Laboratory results

Date	Laboratory result (reference range)														
	Hemoglobin mmol/L (8.5‐10.5)	CRP mg/L (0‐5)	MCV fL (80‐100)	Reticulocytes 10^9^/L (35‐105)	Vitamin B12 pmol/L (150‐700)	Haptoglobin g/L (0.3‐2.0)	Bilirubin total µmol/L (0‐17)	Bilirubin direct µmol/L (0‐7)	LDH U/L (0‐248)	TL µg/ml (3‐7)	ATI AE/mL (<12)	M‐protein g/L	ANA	ANCA	Coombs
29‐3‐2015^a^															
29‐5‐2015	8.7														
16‐7‐2015^b^		3.6													
11‐9‐2015^b^		2.9													
6‐11‐2015^b^		2.9													
12‐1‐2016	6.5														
26‐1‐2016	6.4		106.7	113.7	165							Negative	Positive	Negative	
6‐2‐2016	6														
12‐2‐2016	6.4					<0.2									
17‐2‐2016	6.1														Negative
7‐3‐2016	6.2														
4‐4‐2016	5.4														
15‐4‐2016	5.4		110												
2‐5‐2016	5.6				156		21	8	247						
26‐5‐2016	5.5														
8‐6‐2016	5.5			76.6			27	9	254						
10‐6‐2016^a^	5.4														
14‐7‐2016	5.7		108.2	59.5											
4‐8‐2016^a^	6		107.5	66.3		<0.2									
26‐8‐2016	6.2														
14‐9‐2016	6.5						18	6							
30‐9‐2016^a^	6.7						16								
9‐11‐2016	7.2		106.5	75.4											
25‐11‐2016^a^															
20‐1‐2017^a^															
1‐3‐2017	7.3														
17‐3‐2017^a^															
12‐5‐2017^a^	7.3		97.5							4.2	<12				

a Remicade infusion.

b CT‐P13 infusion.

## 3 DISCUSSION AND CONCLUSIONS

We here describe a 61‐year‐old male CD patient who was diagnosed with hemolytic anemia 6 months after switching from Remicade^®^ to IFX biosimilar CT‐P13. After excluding other causes of progressive symptomatic anemia, a remarkable finding was the relationship in time between the switch from Remicade^®^ to CT‐P13 and the Hb drop. Hence, switching to CT‐P13 was the only intervention in this period, 6‐MP treatment was continued together with all other comedication. It was decided to switch him back to Remicade^®^, which was followed by rapid relieve of his symptoms and an increase in his Hb. The most important problems associated with IFX treatment are immunogenicity (ie, the formation of antidrug antibodies) and autoimmunity. In 2003, an association between anti‐Tumor Necrosis Factor (TNF) treatment and autoimmunity was reported by Vermeire et al^13^ In this cohort, 125 CD patients were prospectively followed and clinical data and autoantibodies were investigated before starting anti‐TNF treatment and at serial time points after IFX initiation. Before start of treatment, the prevalence of ANA was low (7.2%) and after 24 months, 57% of the patients became ANA positive. The majority of these patients (75%) developed ANAs after <3 IFX infusions. In this cohort, one patient with hemolytic anemia was identified 6 months after initiation of IFX treatment. Similar to our patient, the Coombs test was negative.

Although this complication is very rare, switching to CT‐P13 seems to be the most likely cause for the hemolytic anemia. The time relationship together with the improvement after switching back to Remicade^®^ supports this hypothesis.

To the best of our knowledge, we here report for the first time a CD patient who developed a hemolytic anemia after switching from Remicade^®^ to biosimilar IFX. The patient is currently in complete remission while receiving monotherapy with IFX originator. The patient is regularly seen by his treating gastroenterologist for routine follow‐up, including laboratory check‐ups every 4 months.

## CONFLICT OF INTEREST

Anne Strik: no conflicts of interest. Mark Löwenberg**:** has received speaking fees from Abbvie, Covidien, Dr Falk, Ferring Pharmaceuticals, Merck Sharp & Dohme, Receptos, Takesa, Tillotts, and Tramedico. He has received research grants from AbbVie, Merck Sharp & Dohme, Achmea healthcare and ZonMW. Geert D’Haens: has served as advisor for Abbvie, Ablynx, Amakem, AM Pharma, Avaxia, Biogen, Bristol Meiers Squibb, Boerhinger Ingelheim, Celgene, Celltrion, Cosmo, Covidien, Ferring, DrFALK Pharma, Engene, Galapagos, Gilead, Glaxo Smith Kline, Hospira, Immunic, Johnson and Johnson, Lycera, Medimetrics, Millenium/Takeda, Mitsubishi Pharma, Merck Sharp Dome, Mundipharma, Novonordisk, Pfizer, Prometheus laboratories/Nestle, Protagonist, Receptos, Robarts Clinical Trials, Salix, Sandoz, Setpoint, Shire, Teva, Tigenix, Tillotts, Topivert, Versant, and Vifor and received speaker fees from Abbvie, Ferring, Johnson and Johnson, Merck Sharp & Dohme, Mundipharma, Norgine, Pfizer, Shire, Millenium/Takeda, Tillotts, and Vifor.

## AUTHORS CONTRIBUTIONS

Anne Strik (AS) collected and analyzed data and wrote the manuscript. Mark Löwenberg (ML) and Geert D’Haens (GD) contributed to manuscript preparation.
